# Factors affecting physicians using mobile health applications: an empirical study

**DOI:** 10.1186/s12913-021-07339-7

**Published:** 2022-01-04

**Authors:** Pei Wu, Runtong Zhang, Jing Luan, Minghao Zhu

**Affiliations:** Department of Information Management, School of Economics and Management, Beijing Jiaotong University, Beijing, China

**Keywords:** mHealth apps, Physicians usage behavior, Altruism, Cognitive trust, Online rating, UTAUT2

## Abstract

**Background:**

Mobile health applications (mHealth apps) have created innovative service channels for patients with chronic diseases. These innovative service channels require physicians to actively use mHealth apps. However, few studies investigate physicians’ participation in mHealth apps.

**Objective:**

This study aims to empirically explore factors affecting physicians’ usage behaviors of mHealth apps. Based on the extended Unified Theory of Acceptance and Use of Technology (UTAUT2) and mHealth apps features, we propose a research model including altruism, cognitive trust, and online ratings.

**Methods:**

We collected data from physicians who have used mHealth apps and conducted a factor analysis to verify the convergence and discriminative effects. We used a hierarchical regression method to test the path coefficients and statistical significance of our research model. In addition, we adopted bootstrapping approach and further analyzed the mediating effects of behavioral intention between all antecedent variables and physicians’ usage behavior. Finally, we conducted three robustness analyses to test the validity of results and tested the constructs to verify the common method bias.

**Results:**

Our results support the effects of performance expectancy, effort expectancy, social influence, and altruism on the behavioral intentions of physicians using mHealth apps. Moreover, facilitating conditions and habits positively affect physicians using mHealth apps through the mediating effort of behavioral intention. Physicians’ cognitive trust and online rating have significant effects on their usage behaviors through the mediating efforts of behavioral intention.

**Conclusions:**

This study contributes to the existing literature on UTAUT2 extension of physicians’ acceptance of mHealth apps by adding altruism, cognitive trust, and online ratings. The results of this study provide a novel perspective in understanding the factors affecting physicians’ usage behaviors on mHealth apps in China and provide such apps’ managers with an insight into the promotion of physicians’ active acceptance and usage behaviors.

**Supplementary Information:**

The online version contains supplementary material available at 10.1186/s12913-021-07339-7.

## Background

Mobile phones and smartphones have become an important part of daily life and the widespread use of these mobile devices has contributed to the convergence of healthcare services and mobile technologies [1]. Mobile health (mHealth) refers to a type of health service applied to mobile computing, medical sensors, and communication technology [2, 3]. In 2015, more than 165,000 mHealth apps were available for users, and most mHealth apps focused on health management (65%), and disease and treatment management (24%). Participants of mHealth apps include patients and professional physicians, but prior studies little consider physicians’ acceptance and usage behaviors of mHealth apps [4]. Different types of users, including patients and physicians, can easily publish and access health information anytime and anywhere through mHealth apps [5, 6]. Physicians through mHealth apps access medical literature, quickly collect patients’ disease information, and communicate with patients in real-time. For patients, mHealth apps provide rich health services and information for them and they need to find information of high value to promote their health and well-being [7, 8]. Patients who conduct health self-management with physicians’ guidance can easily enable patient-physician partnerships and empower their healthcare by integrating professional knowledge and other patients with similar diseases experiences [9, 10]. The lack of physicians’ participation may limit the valuable use and success of mHealth apps. Therefore, Exploring factors on physicians’ acceptance of mHealth apps is meaningful and greatly improves the effective engagement with mHealth apps [11].

The topic of mHealth apps in recent years has become the focus in the field of healthcare informatics. For example, research on examining the influence of patients’ opinions on physician quality [12], the antecedent variables affecting patients’ acceptances of mHealth services in developing countries [13], and the recommendation of mHealth apps based on behavior change techniques [14]. Though most studies have explored the effect of behavioral intentions and usage behaviors of mHealth services [13, 15], the literature has not yet addressed the issue of mHealth apps adoption from the perspectives of specific types of users. To address this research gap, this study focuses on physician-centric mHealth apps, a form of mobile platform maintained by physicians, in which patients can consult with physicians and other patients who have consulted with the same physicians [9]. We aimed to examine the antecedent factors affecting physicians’ usage behaviors of mHealth apps. In this study, usage behaviors refer to physicians’ ongoing and everyday post-acceptance use of mHealth apps. Based on UTAUT2, we propose a research model to predict physicians’ intentions and behaviors of mHealth app usage. The contribution of this study is that the research model innovatively added altruism, cognitive trust, and online rating in the research model based on the features of mHealth apps. Altruism reflects personal social responsibility and mission [16]. Physicians take altruism as the basic ethics and work in the best interests of patients [17]. Compared with nonmedical service providers, physicians are more willing to sacrifice their benefits to promote patients’ healthcare outcomes [8]. Cognitive trust reflects the ability of mHealth apps to provide information reliably, safely, and accurately [18], which plays a critical role in encouraging physicians to adopt the emerging platforms of health information and services provided. Online ratings are the innovative feature of mobile technology. Online ratings published through mHealth apps reflect the overall evaluation related to online health services of physicians by patients that maybe affect physicians’ intention and behavior of usage of such apps [19]. From the perspective of physicians, online ratings belong to an intrinsic motivation to use mHealth apps, and physicians who obtain high online ratings have a higher sense of self-worth.

### Mobile health applications

Mobile technology adoption is an important exploration in the current fields of information systems. Prior studies have explored various factors that affect the acceptance of information technologies [20]. Recently, studies focusing on mHealth have grown rapidly and the value of mHealth based on mobile technologies has gradually been recognized [21, 22]. The use of mobile phones and the development of mHealth apps have aroused the interest of researchers in information systems [23]. Mohammad used the UTAUT2 theoretical framework to study the factors promoting the adoption of mHealth services in the developing country and examined the moderating effects of gender on usage intention and behavior [21]. In addition, Murnane et al. classified mHealth apps at a fine-grained level and examined the perceived efficacy and the factors of potential adoption and abandonment of such apps [24]. Krebs and Duncan investigated the use of mHealth apps in the United States and found that most people did not use mHealth apps and even among those who used mHealth apps at first stopped using such apps, which found that the main reasons may be the heavy burden of information input, loss of interest and potential costs [4]. Based on the above literature, we found that prior studies focused on users’ behaviors in mHealth apps and rarely discussed physicians’ usage behavior in mHealth apps. The guidance of physicians to patients in mHealth apps plays a vital role in patients’ self-management. In contrast to prior studies, this study focuses on the characteristics of physicians using mHealth apps in China and combined with the theoretical framework of UTAUT2, which is widely used in the field of information systems acceptance [24], to explore physicians using behaviors.

### Theoretical background

UTAUT is the most comprehensive theory in the field of information systems and is used to understand the acceptance of information technology in various environments [25]. UTAUT assumes that antecedents (performance expectations, effort expectations, social influence) indirectly affect usage behaviors through behavioral intentions, behavioral intentions and facilitating conditions directly affect usage behaviors, and the moderation effects of factors (gender, age, experience, and voluntariness of use) on the relationships between antecedents and usage behaviors [26]. UTAUT2 was proposed which has been widely used in technical user scenarios and expanded the three external constructs of hedonic motivation, price value, and habit into UTAUT [27]. Existing studies have expanded UTAUT2 to different types of users in explaining the process of their technology acceptance. For example, Chuah et al. discussed consumers’ adoption of smartwatches based on UTAUT2 and other dominant technology adoption theories [28]. Wang et al. used UTAUT2 to verify the habitual behavior of Chinese on social media [29]. For special types of users, Stefi et al. explored the influence of reviewing software developers’ reuse of software components in the organization based on UTAUT2 [30]. In addition, Escobar et al. examined the driving factors behind different types of consumer purchases of air tickets, including examining consumer users types and citizen user types [31]. Dwivedi et al. investigated the factors influencing the adoption of mHealth by citizens from different countries [32]. However, previous studies rarely have focused on specific types of user categories, such as teachers, students, tourists, and jobseekers.

**Fig. 1:**
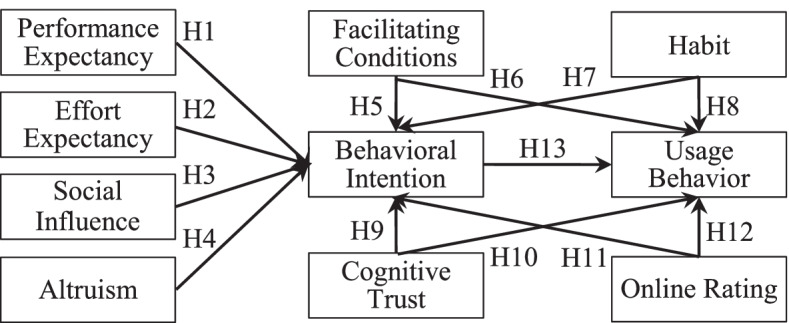
Research model

To extend previous studies, this study focuses on the physicians using mHealth apps based on UTAUT2. In the context of physicians, hedonic motivation is not the main factor affecting Chinese physicians to use mHealth apps as they have heavy offline work. Thus, this study removed the constructs of hedonic motivation from UTAUT2 and added altruism as physicians’ intrinsic motivation. In addition, considering that physicians have health expertise and high judgment on the professionalism of mHealth apps, we speculate that physicians’ cognitive trust in mHealth apps affects usage behavior because cognitive trust has been proved to be closely related to individuals’ perceived competence [33]. Finally, physicians’ online ratings provided by patients who have consulted health services on mHealth apps may encourage physicians to actively use such apps. Online ratings also reflect physicians’ activity in mHealth apps, which improves physicians’ online social value and attracts other physicians to participate in such apps for communication and health knowledge interaction. Our proposed research model is presented in Fig. 1.

### Hypotheses development

#### Performance expectancy

People expect that using emerging technologies will help them enhance their job performance [19]. Performance expectancy is an important variable of peoples’ behavioral intentions and has been proved to significantly affect individuals’ intention to accept emerging information technologies [26, 34–36]. Using smartphones has been proved to be convenient for people to improve their work performance [37]. Performance expectancy represents physicians’ beliefs about mHealth apps before use, and these beliefs may affect physicians’ behavioral intentions [26]. Thus, we propose the following hypothesis:

- *H1: Performance expectancy positively affects physicians’ usage intentions.*

#### Effort expectancy

People expect the process of using emerging technologies to be simple and easy. Effort expectancy is a construct about how easy it is to use emerging technologies in prior studies on technology acceptance [26]. Perceived ease of use significantly affects behavioral intentions [38]. With the increase in effort expectancy, emerging technology usage is believed to require minimal effort [39]. Physicians’ behavioral intentions of using mHealth apps may be related to effort expectancy. Thus, we propose the following hypothesis:

- *H2: Effort expectancy positively affects physicians’ usage intentions.*

#### Social influence

The opinions of some friends and relatives opinions on the acceptance of emerging technology will influence individuals’ behavioral intentions, which is the definition of social influence [40]. Social influence reflects how individuals’ behaviors of mHealth app usage are influenced by others’ opinions [38]. Most physicians are unfamiliar with mHealth apps that are emerging applications and lack time to participate in actives through such apps [41], so physicians’ intentions of usage mHealth apps may tend to rely on others’ perceptions. Thus, we propose the following hypothesis:

- *H3: Social influence positively affects physicians’ usage intentions.*

#### Altruism

Altruism is when people help others without expecting anything in return [42]. Physicians help others voluntarily and selflessly, gaining happiness by showing altruism [43]. Different from patients of mHealth apps, physicians are more willing to help patients at the expense of their interests and enjoy helping patients solve health problems and answer patients’ doubts [44, 45]. Physicians as health professionals instinctively master altruism and work for the best interests of patients [17]. As an intrinsic motivation, altruism plays a significant role in determining emerging technologies’ adoption and usage [46]. In the original model in UTAUT2, the intrinsic motivation of technical users to accept the use of information systems in hedonic motivation [27]. The intrinsic motivation of physicians to participate in mHealth apps is not hedonic motivation. This study supplements intrinsic motivation for altruism to help understand physicians’ intention to use mHealth apps. Thus, we propose the following hypothesis:

- *H4: Altruism positively affects physicians’ usage intentions.*

#### Facilitating condition

Facilitating conditions are defined as the extent to which people believe that potential conditions, such as technical infrastructure, human support, and compatibility, exist to support the use of an emerging technology [26]. In the context of technical infrastructure, objective conditions in mHealth apps believed by physicians make the operation easy, including the provision of mobile devices support. In the context of human support, the guidance of mHealth apps is available to physicians in the process of decision-making of user behavior. In the context of compatibility, most physicians are highly educated professionals with the ability to use mHealth apps. The higher the level of hospital support, the more favorable the mHealth app usage is among physicians [47]. Prior studies have found significant relationships between facilitating conditions and usage intentions and behaviors of healthcare information systems [48, 49]. Thus, we propose the following hypothesis:

- *H5: Facilitating condition positively affects physicians’ usage intentions.*
- *H6: Facilitating condition positively affects physicians’ usage behaviors of mHealth apps.*

#### Habit

Habit refers to the individuals’ tendencies to behaviors based on cumulative learning experience [50]. The results of accumulated learning experiences and habitual behavior might affect individuals’ attitudes and beliefs, which have a significant effect on intentions and usage behaviors [51]. The effects of habits on intentions and actual usage behaviors are reflected in the mobile apps field [52, 53], such as mobile payments and mobile banking services. In the health field, there may be a significant correlation between habit and physicians’ intentions and use behaviors of such apps. Thus, we propose the following hypothesis:

- *H7: Habit positively affects physicians’ usage intentions.*
- *H8: Habit positively affects physicians’ usage behaviors of mHealth apps.*

#### Cognitive trust

Cognitive trust refers to users’ perception confidence in the accurate functions promised by emerging technology providers when using technical processes [54]. Physicians provide health services and information for patients through mHealth apps. If physicians are unable to operate the mHealth apps comfortably, they will become frustrated and consider mHealth apps to be unreliable [55]. Previous studies proposed that people perceive information systems as trustworthy due to their user-friendliness [56]. In addition, cognitive trust is related to physicians’ competence and has been confirmed to affect behavioral intention and use behavior of emerging technologies [57, 58]. When physicians believe that mHealth apps are reliable and help to provide health services, they may be inclined to trust such apps. Physicians’ intentions and behaviors of using mHealth apps may be strengthened due to cognitive trust. Thus, we propose the following hypothesis:

- *H9: Cognitive trust positively affects physicians’ usage intentions.*
- *H10: Cognitive trust positively affects physicians’ usage behaviors of mHealth apps.*

#### Online rating

Information exchange through mHealth apps allows patients to publish ratings on physicians and share ratings with other patients on such apps [59]. Online rating objectively reflects the average evaluation of patients on the health services provided by physicians. Online ratings, the innovative feature of mobile technology, can motivate physicians to actively use behaviors and more easily help physicians evaluate the experience of using mHealth apps [19, 60]. Prior studies found that a significant correlation between online ratings’ features and individuals’ behaviors [61, 62]. Thus, we propose the following hypothesis:

- *H11: Online rating positively affects physicians’ usage intentions.*
- *H12: Online rating positively affects physicians’ usage behaviors of mHealth apps.*

#### Behavioral intention and usage behavior

Intentions play an important role in predicting usage behaviors of emerging technologies. Prior studies have confirmed that behavioral intentions are highly related to user behavior and behavioral intention is the main factor in using mobile services [14]. In this study, intentions are considered the extent to which physicians perceive their willingness to use mHealth apps. Usage behaviors are considered the ongoing and everyday post-acceptance use of mHealth apps. We expect that physicians’ behavioral intentions to use mHealth apps may positively affect their usage behaviors. Thus, we propose the following hypothesis:

- *H13: Physicians’ intentions positively affect their usage behaviors of mHealth apps.*

## Methods

### Measurement

To evaluate the research model, this study adopted valid scales in prior studies and modified all scales to adapt to the context of mHealth apps. As all participants were Chinese, we invited two researchers who were familiar with English and Chinese to translate all English items into Chinese and back translate Chinese items to English. We adopted a back-translation method to minimize language differences [63]. In addition, our questionnaire was verified by five experts in the information technology adoption field. In the pilot study, 20 physicians from different hospitals were recruited to fill in our questionnaire. We made amendments based on feedback in the pilot study and determined the final version of our questionnaire.

As seen in Additional file 1, performance expectancy, effort expectancy, social influence, facilitating conditions, habit, behavioral intention, and usage behaviors were validated using items extracted from Venkatesh et al. [27]. Cognitive trust was validated using scale items extracted from Lu et al. [58]. Items of online rating came from Alalwan [34] and Filieri [60]. Altruism was validated using items extracted from Zhang et al. [8]. We used a multiple-item scale with a seven-point Likert-type replied scheme anchored at “1 = strongly disagree” to “7 = strongly agree” to assess all constructs in the research model. Our questionnaire consisted of three parts, including 42 items and 4 demographic questions. First, the questionnaire presented our purpose and clarifies mHealth apps with three examples (Haodaifuzaixian, Dingxiangyuan, and Chunyuyisheng). Second, we provided some demographic issues specifically. Finally, we introduced all items of our constructs.

### Data collection and sample

We carried out cross-sectional research in two Chinese hospitals from June to July 2020 and the formal investigation was anonymous. Our formal investigation was carried out anonymously in two Chinese hospitals. All samples were randomly selected physicians who had used mHealth apps. To ensure the minimize nonresponse bias and the sample represented the Chinese physicians’ census in terms of gender, age, education, and experience using mHealth apps, we closely worked with the two hospitals and conducted a short introduction and instruction for participations before investigation [64]. In addition, 5 RMB was set as a reward for all respondents’ time and efforts. We guaranteed that all participants had sufficient time to answer all questions and had the right to withdraw from our survey [15]. We distributed 450 questionnaires and received 418 questionnaires, resulting in a 92.9% response rate. In addition, after the survey is administered, all non-respondents received second to complete the survey. Through careful inspection, we deleted incomplete and controversial responses. Finally, we obtained 393 valid responses for analysis.

### Descriptive statistics

The descriptive statistics of 393 respondents are shown in Table 1. We verified whether there are group differences among the physicians in different hospitals through conducting independent-sample T-tests. The results showed that no significant differences in control variables in our study. As shown in Table 1, more than half of the respondents were female. About 50% of the respondents were 18-29 years old, and only 13% of the respondents were over 40 years old. The results reflect that young physicians were the main members of mHealth apps. In addition, the majority of respondents were university graduates (74%). Most of the respondents have used mHealth apps within 1 year, and the number of respondents with more than 3 years of experience using mHealth apps was very small, which indicated that mHealth apps are emerging for physicians in China.

**Table 1:** Demographic profiles of samples (*N* = 393)

Demographic profile	Criteria	Frequency	Percentage (%)
Gender	Male	182	46.3
	Female	211	53.7
Age	18-29	198	50.4
	30-40	144	36.6
	40 and above	51	13.0
Education level	High school and below	30	7.6
	Graduate	291	74.1
	Postgraduate and above	72	18.3
Experience using mHealth apps	1 years and below	292	74.3
	1 to 3 years	82	20.9
	3 years and above	19	4.8

## Results

### Measurement testing

To test the measurement model, we first conducted a factor analysis to verify the convergence and discriminative effects of all items following the Kaiser criterion (significant components have eigenvalues over 1.0). Moreover, the value of Kaiser-Meyer-Olkin (KMO) was 94.5%, and the collected data were acceptable [65] and fit for confirmatory factor analysis [66]. Next, we conducted a confirmatory factor analysis to examine the relationships between factors and measurement items. The results of factor loadings were shown in Table 2. We examined the reliability, content validity, and structural validity of each construct. Given that all the items were based on existing studies, we ensured our questionnaire’s content effectiveness. Cronbach’s alpha and the composite reliability (CR) were over 0.7, which supported the reliability of constructs [65]. Moreover, the factor loadings of all items were over 0.7, and the average variance extracted (AVE) values exceeded 0.5, which indicated that our scales’ convergent validity was acceptable [65].

**Table 2:** Results of Construct Validity and Reliability

Constructs	Items	Mean	S.D.	Factor loading	Cronbach’s alpha	CR	AVE
Performance expectancy (PE)	PE1	5.776	1.062	.769	.790	.819	.531
	PE2	5.260	1.201	.701			
	PE3	5.542	1.195	.716			
	PE4	5.407	1.279	.727			
Effort expectancy (EE)	EE1	5.705	1.241	.793	.779	.849	.584
	EE2	5.102	1.307	.737			
	EE3	5.618	1.221	.758			
	EE4	5.628	1.122	.768			
Social influence (SI)	SI1	5.056	1.333	.821	.816	.885	.658
	SI2	4.972	1.278	.783			
	SI3	5.021	1.314	.777			
	SI4	4.336	1.476	.860			
Altruism (AL)	AL1	5.501	1.167	.778	.808	.843	.574
	AL2	5.372	1.156	.755			
	AL3	5.415	1.184	.759			
Facilitating conditions (FC)	FC1	5.519	1.441	.783	.752	.881	.597
	FC2	5.476	1.280	.836			
	FC3	5.328	1.221	.735			
	FC4	5.557	1.186	.773			
Habit (HB)	HB1	4.946	1.417	.798	.839	.885	.659
	HB2	4.083	1.656	.878			
	HB3	4.038	1.619	.750			
	HB4	4.831	1.439	.817			
Cognitive trust (CT)	CT1	5.382	1.177	.734	.703	.849	.585
	CT2	4.804	1.466	.777			
	CT3	4.835	1.454	.759			
	CT4	5.453	1.056	.789			
Online rating (ORT)	ORT1	5.148	1.245	.797	.864	.893	.544
	ORT2	5.142	1.231	.704			
	ORT3	4.819	1.321	.769			
	ORT4	5.216	1.194	.725			
	ORT5	5.066	1.219	.744			
	ORT6	5.043	1.319	.716			
	ORT7	5.438	1.225	.705			
Behavioral intention (BI)	BI1	5.718	1.122	.740	.811	.784	.547
	BI2	5.651	1.188	.755			
	BI3	5.511	1.262	.724			
Usage Behavior (UB)	UB1	5.356	1.208	.701	.752	.776	.536
	UB2	5.183	1.316	.740			
	UB3	5.300	1.264	.755			

*Note: CR* Composite Reliability, *AVE* Average Variance Extracted, *PE* Performance Expectancy, *EE* Effort Expectancy, *SI* Social Influence, *AL* Altruism, *FC* Facilitating Conditions, *HB* Habit, *CT* Cognitive Trust, *ORT* Online Rating, *BI* Behavioral Intention, *UB* Usage Behavior

Second, we measured the constructs’ relationships. Table 3 shows that the square root of AVE exceeded all correlation coefficients between arbitrary constructs, which indicated that the discriminant validity of all constructs was acceptable [67]. We also evaluated goodness-of-fit indexes to test configural invariance and metric invariance based on the evaluation criteria [68]. As shown in Table 4, the results reveal strong support for measurement invariance.

**Table 3:** Discriminant Validity

Constructs	PE	EE	SI	AL	FC	HB	CT	ORT	BI	UB
Performance expectancy (PE)	**.729**									
Effort expectancy (EE)	.433	**.764**								
Social influence (SI)	.505	.442	**.811**							
Altruism (AL)	.599	.576	.636	**.758**						
Facilitating conditions (FC)	.474	.600	.483	.583	**.773**					
Habit (HB)	.533	.486	.744	.699	.579	**.812**				
Cognitive trust (CT)	.207	.153	.325	.241	.212	.308	**.765**			
Online rating (ORT)	.500	.498	.606	.712	.520	.752	.244	**.738**		
Behavioral intention (BI)	.542	.600	.592	.694	.569	.677	.217	.603	**.740**	
Usage Behavior (UB)	.552	.504	.749	.620	.545	.800	.266	.620	.642	**.732**

*Note:* Diagonal values are squared roots of AVE; Off-diagonal values are the estimates of inter-correlation between the latent constructs; *PE* Performance Expectancy, *EE* Effort Expectancy, *SI* Social Influence, *AL* Altruism, *FC* Facilitating Conditions, *HB* Habit, *CT* Cognitive Trust, *ORT* Online Rating, *BI* Behavioral Intention, *UB* Usage Behavior

**Table 4:** Goodness of fit assessments for the research model

Goodness of fit measures	CMIN/DF	IFI	TLI	CFI	GFI	RMSEA	SRMR
Goodness of fit ranges	1-3	> .900	> .900	> .900	> .900	< .050	< .050
Measurement model	1.377	.938	.930	.940	.895	.041	.042

*Note: CMIN/DF* Chi square/Degrees of freedom, *IFI* Incremental fit index, *TLI* Tucker-Lewis index, *CFI* Comparative fit index, *GFI* Goodness of fit index, *RMSEA* Root-mean-square error of approximation, *SRMR* Standardized root-mean-square residual

### Hypothesis testing

To verify our hypothesis, we used a hierarchical regression method to test the path coefficients and statistical significance of our research model.

1a $${BI}_i={\alpha}_0+{\alpha}_1\times GEN+{\alpha}_2\times AGE+{\alpha}_3\times EDU+{\alpha}_4\times EXP+{\delta}_i$$

1b $${BI}_i={\alpha}_0+{\alpha}_1\times GEN+{\alpha}_2\times AGE+{\alpha}_3\times EDU+{\alpha}_4\times EXP+{\alpha}_5\times PE+{\alpha}_6\times EE+{\alpha}_7\times SI+{\alpha}_8\times HM+{\alpha}_9\times FC+{\alpha}_{10}\times HB+{\alpha}_{11}\times CT+{\alpha}_{12}\times ORE+{\delta}_i$$

2a $${BI}_i={\beta}_0+{\beta}_1\times GEN+{\beta}_2\times AGE+{\beta}_3\times EDU+{\beta}_4\times EXP+{\varepsilon}_i$$

2b $${UB}_i={\beta}_0+{\beta}_1\times GEN+{\beta}_2\times AGE+{\beta}_3\times EDU+{\beta}_4\times EXP+{\beta}_5\times FC+{\beta}_6\times HB+{\beta}_7\times CT+{\beta}_8\times ORE+{\beta}_9\times BI+{\varepsilon}_i$$

Where *α*_*i*_ and *β*_*j*_ are the coefficients to be estimated. *δ*_*i*_ and *ε*_*j*_ represent the random errorterm.

Our hypotheses were tested based on the above two models. The dependent variable of the model (1) is physicians’ behavioral intention. First, control variables are added to model 1a, including gender, age, education level, and the length of time using mHealth apps; Second, model 1b added the independent variables based on model 1a. Similarly, the dependent variable of the model (2) is physicians’ usage behavior. First, control variables are added to model 2a; Second, model 2b added the independent variables based on model 2a. The results were listed in Table 5.

**Table 5:** Results of the Hypothesis Testing

Dependent Variable		Model 1a		Model 1b		Model 2a		Model 2b	
		S.B.	S.E.	S.B.	S.E.	S.B.	S.E.	S.B.	S.E.
Constant		6.516^***^	.353	1.940^***^	.380	5.813^***^	.411	1.657^**^	.521
Control Variable	GEN	−.205	.113	−.208	.083	−.145	.131	−.042	.111
	AGE	−.177^*^	.083	.069	.060	−.157	.096	−.123	.080
	EDU	.316^**^	.112	−.190	.082	−.412^**^	.131	−.323^**^	.110
	EXP	.361^**^	.103	−.056	.078	.549^***^	.120	.145	.104
Independent Variable	PE			.186^***^	.049				
	EE			.175^***^	.038				
	SI			.110^**^	.041				
	AL			.217^***^	.050				
	FC			.096^*^	.038			.154^**^	.048
	HB			.207^***^	.035			.256^***^	.047
	CT			.113^*^	.046			.131^**^	.046
	ORT			.116^*^	.043			.127^*^	.051
	BI							.322^***^	.052
R-squared		.055		.523		.071		.495	
Adjusted R-squared		.045		.508		.061		.478	
F-statistics		5.632^***^		34.742^***^		7.365^***^		20.472^***^	

Note:^***^p < 0.001, ^**^p < 0.01, ^*^p < 0.05; *GEN* Gender, *AGE* Age, *EDU* Education Level, *EXP* Experience using mHealth apps, *PE* Performance Expectancy, *EE* Effort Expectancy, *SI* Social Influence, *AL* Altruism, *FC* Facilitating Conditions, *HB* Habit, *CT* Cognitive Trust, *ORT* Online Rating, *BI* Behavioral Intention, *UB* Usage Behavior

The results show that performance expectancy, effort expectancy, social influence, and altruism positively affected intentions of using mHealth apps, so H1, H2, H3, and H4 were supported. As hypothesized, facilitating conditions showed positive associations with physicians’ behavioral intention and behavior intention, so H5 and H6 were supported. Moreover, the relationships between physicians’ habits and usage behavior, and between their usage intentions and behaviors were remarkable. Thus, H8 and H13 were supported. In addition, habit had significant effects on physicians’ intentions of using mHealth apps. Thus, H7 was supported. In this study, the significant positive effects of online ratings on use behavior were also found. Thus, H12 was supported.

### Mediating effect testing

We adopted bootstrapping approach and further analyzed the mediating effects of behavioral intention between all antecedent variables and physicians’ usage behavior. The bootstrapping method is not restricted by the normality assumption of statistics and is the unbiased estimation which is more suitable than other statistical methods, such as the Sobel test, to test the mediation effects. Thus, we adopted bootstrapping test and the criterion is that if 0 is not included in the 95% confidence interval of the total effects, direct effects, and indirect effects, then the mediating effect is significant [69, 70]. As shown in Table 6, the 95% confidence intervals for all mediation effects do not include 0, which indicated physicians’ behavioral intention has a significant mediation effect in the context of mHealth apps usage and extend UTAUT2.

**Table 6:** Bootstrapping analysis of the mediation effects

Effects	Dependent Variable		Effects	Boot SE	Bootstrap 95%CI	
					Boot LLCI	Boot ULCI
Total Effects	Independent Variable	PE	.498	.003	.316	.472
		EE	.301	.047	.209	.393
		SI	.426	.041	.346	.506
		AL	.504	.047	.412	.595
		FC	.203	.042	.121	.286
		HB	.406	.040	.328	.483
		CT	.170	.052	.068	.272
		ORT	.323	.047	.216	.416
Indirect Effects	Independent Variable	PE	.217	.074	.413	.705
		EE	.194	.038	.123	.273
		SI	.131	.030	.239	.438
		AL	.188	.046	.104	.281
		FC	.136	.028	.086	.194
		HB	.153	.034	.090	.221
		CT	.064	.024	.019	.115
		ORT	.189	.040	.117	.275
Direct Effects	Independent Variable	PE	.281	.081	.138	.455
		EE	.107	.054	.007	.218
		SI	.295	.053	.191	.397
		AL	.316	.075	.163	.462
		FC	.067	.044	−.012	.146
		HB	.253	.052	.152	.354
		CT	.106	.051	.010	.211
		ORT	.134	.060	.016	.256

*Note: PE* Performance Expectancy, *EE* Effort Expectancy, *SI* Social Influence, *AL* Altruism, *FC* Facilitating Conditions, *HB* Habit, *CT* Cognitive Trust, *ORT* Online Rating, *BI* Behavioral Intention, *UB* Usage Behavior

### Robustness check

**Fig. 2:**
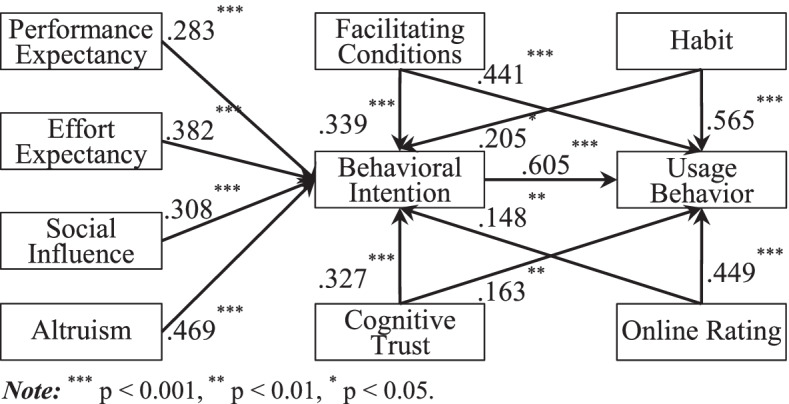
Path coefficients and significance levels. Note:^***^p < 0.001, ^**^p < 0.01, ^*^p < 0.05

To test the validity of our results, we conducted three robustness analyses. First, we examined the error terms of our independent variables. The results showed that the Shapiro-Wilk test, w-value, and *p*-value are not significant. Thus, all independent variables’ error terms obey normal distribution. Next, we applied an algorithm [70] to test lower bounds on sample size in our research model. We calculated all samples’ statistical powers through GPower. The results showed that the statistical power of our research model is above 0.8. Thus, our sample size could explain the research model. Finally, we carried out another analysis based on the partial least squares method [71], and the results were listed in Table 7 and Fig. 2. These results were consistent with the systems of regression equations. In addition, the research model could explain variance in physicians’ intentions with 0.674 and usage behaviors with 0.981. Prior studies proved that UTAUT2 constructs could explain variance in intentions with 0.541 and usage behaviors with 0.891. The addition of cognitive trust and online rating to our proposed research model improved the predictive validity, thereby laying a theoretical foundation for this study.

**Table 7:** SEM results of the Hypothesis Testing

Hypothesis	Relationship	Std. Beta	Std. Error	***t***-value	Result
H1	PE → BI	.283***	.086	3.271	Support
H2	EE → BI	.382***	.079	4.783	Support
H3	SI → BI	.308***	.047	6.080	Support
H4	AL → BI	.469***	.098	4.804	Support
H5	FC → BI	.339***	.093	3.622	Support
H6	FC → UB	.441***	.075	5.901	Support
H7	HB → BI	.205*	.084	2.469	Support
H8	HB → UB	.565***	.069	8.250	Support
H9	CT → BI	.327***	.053	6.170	Support
H10	CT → UB	.163**	.063	2.577	Support
H11	ORT → BI	.148**	.057	2.596	Support
H12	ORT → UB	.449***	.077	5.841	Support
H13	BI → UB	.605***	.094	6.436	Support

Note:^***^p < 0.001, ^**^p < 0.01, ^*^p < 0.05; *PE* Performance Expectancy, *EE* Effort Expectancy, *SI* Social Influence, *AL* Altruism, *FC* Facilitating Conditions, *HB* Habit, *CT* Cognitive Trust, *ORT* Online Rating, *BI* Behavioral Intention, *UB* Usage Behavior

### Common method bias

We tested the constructs to verify the common method bias. First, we carried out Harmon’s one-factor test [72, 73]. The results show that the single factor could explain 27% of the total variance. Second, we tested the measurement model by adding a latent common method variance factor [74]. The results show that the path coefficients and significance levels of our proposed measurement model are stable. All results show that there is no threat to the common method bias.

## Discussion

Our results supported most antecedent variables to predict physicians’ intentions and usage behaviors of mHealth apps. All constructs’ reliability and validity reached acceptable standards and we proved the goodness of fit of our measurement model to the collected data through confirmatory factor analysis. Our research model confirmed the importance of including cognitive trust and online rating to UTAUT2 in the context of physicians’ use of mHealth apps in China, and the path coefficients proved our hypotheses. Physicians who use mHealth apps can more save time and effort when providing mHealth services to patients. Physicians’ performance expectancy significantly affected their behavioral intention of using mHealth apps, and such results were parallel with those reached by prior studies concerning mHealth services [15, 21].

Facilitating conditions were found to be a crucial construct on behavioral intentions. Physicians are willing to use mHealth apps if they feel comfortable and reliable. In the context of physicians, the reason for our results may be that, facilitating conditions and features are sufficient on their own to guarantee physicians’ behavioral intention [34]. Technical and human support is a significant issue for physicians who are willing to use mHealth apps. We also found a positive effect of habit on physicians’ usage behaviors. Thus, physicians who are likely to have a habitual behavior of adopting emerging technologies are willing to use mHealth apps. The results confirmed the significant effects of cognitive trust and online rating on physicians’ intentions of using mHealth apps. We believe that physicians might be interested in online ratings provided by patients in mHealth apps as online ratings may belong to physicians’ intrinsic motivation to use mHealth apps. Physicians who obtain high online ratings have a higher sense of self-worth and positive participant in online health services on mHealth apps.

### Theoretical implications

Our research has several significant theoretical implications on physicians’ acceptance of mHealth apps in China. First, this study improves the understanding of physicians’ behaviors by focusing on physicians using mHealth apps. Prior studies have scarcely investigated the specific group of physicians. In addition, mHealth apps have several unique characteristics compared with apps that provide electronic commerce services. These unique characteristics determine the intention and behaviors of physicians using mHealth apps.

Second, this study is one of the first studies that consider physicians’ intrinsic motivations, such as altruism, cognitive trust, and online ratings as antecedent variables that influence physicians’ behavioral intention to use mHealth apps. Prior studies have rarely investigated the influence of physicians’ intrinsic motivations on the acceptance of mHealth apps. This study found that physicians’ intrinsic motivations have positive and significant impacts on using intention and behaviors.

Finally, we extended UTAUT2 in information communication technologies (smartphones) and specific users (physicians), which enriches the literature on UTAUT2 application scenarios. This study enriches the literature on physicians’ usage behaviors of mHealth apps in developing countries and reveals key factors in physicians’ intentions and behaviors to use mHealth apps.

### Managerial implications

Our results also provide some managerial implications for physicians using mHealth apps in China. Particularly, the antecedent variables proposed in the research model have been proved to have a significant impact on physicians using mHealth apps. First, performance expectance, effort expectance, and social influence have significant impacts on physicians using mHealth apps. Thus, the administrator of mHealth apps may be able to make the settings more convenient for physicians to use and help physicians achieve higher work performance. When improving physicians’ work performance, managers expand the scope of physicians users, reward physicians who invite others to participate, encourage physicians to advertise to physicians who have used them, and enhance the social influence of mHealth apps. Managers should strive to motivate more physicians to actively participate in mHealth apps.

Second, this study found facilitating conditions and habits have positive and significant effects on physicians’ usage behavior through the mediating effect of physicians using mHealth apps intention. Thus, managers should provide technical support and consultants to physicians who use such apps for the first time and help physicians to guide and operate in mHealth apps. In addition, managers can provide physicians with more attractive help to make them use mHealth apps as a habit, which will encourage physicians to use mHealth apps.

Finally, this study found that cognitive trust and online ratings have significant and positive effects on physicians’ usage behaviors through the mediating effect of usage intention. Thus, the function of mHealth apps should attract physicians’ cognitive trust. The managers of mHealth apps should devote efforts to improving physicians’ cognitive trust and online rating to attract physicians to participate in mHealth apps. For example, managers can provide objective and detailed online ratings of physicians and convince physicians that the online rating plays an important role in the realization of their self-worth. In addition, managers should try to satisfy physicians’ relevance and credibility of online ratings. Managers should ensure that the credibility and reliability of online ratings, which is important to physicians, otherwise physicians may abandon the use of mHealth apps.

### Limitations

This study also had some limitations. First, our survey subjects were physicians who were randomly selected in Chinese hospitals. Given that most users in mHealth apps are patients and physicians, our results may not reflect patients’ acceptance of such apps. Moreover, depending on the characteristics of mHealth apps, there may be different effects of antecedents in our research model on physicians’ usage behaviors in the different mHealth apps. Exploring the difference in physicians’ usage behavior between the different mHealth apps is the future research direction. Second, we found that cognitive trust and online rating had significant effects on physicians’ intentions and behaviors of using mHealth apps, which has not been confirmed by prior studies. Thus, future studies might further investigate the essential effects of other constructs and our results should be further validated in other developing countries and other emerging technologies acceptance. Finally, our research was a cross-sectional study. The initial empirical study using UTAUT2 was longitudinal, so other research on mHealth apps acceptance should be further examined through longitudinal research. In further research, the limitations and effectiveness of UTAUT2 should be checked in mHealth apps.

## Conclusion

This study proposes a research model integrating altruism, cognitive trust, and online rating into UTAUT2 to explore physicians’ intentions and behaviors of using mHealth apps. The results proved physicians’ performance expectancy, effort expectancy, social influence, and facilitating condition have a positive impact on behavioral intention of using mHealth apps. In addition, Facilitating conditions and habit directly affect physicians’ usage behaviors. This study contributes to the existing literature on UTAUT2 extension of physicians’ acceptance of mHealth apps by adding altruism, cognitive trust, and online ratings. The results of this study provide a novel perspective in understanding the factors affecting physicians’ acceptance of mHealth apps in China. This study is of great significance to physicians’ acceptance of mHealth apps and mHealth apps should be integrated into the national health systems to further improve physicians’ usage intentions and behaviors.

## Supplementary Information

**Additional file 1.**

## Data Availability

The authors confirm that the datasets used and/or analysed during the current study available from the corresponding author on reasonable request.
