# Supplementary material for: Factors affecting physicians using mobile health applications: an empirical study
Source: BMC Health Serv Res. 2022 Jan 4;22:24. doi: 10.1186/s12913-021-07339-7 (PMC8729011; doi:10.1186/s12913-021-07339-7)
Supplement: Supplementary file 1 — Additional file 1. [file 12913_2021_7339_MOESM1_ESM.pdf]

Appendix A. Measurement scale and items.

Constructs	Items	Statement	Sources
Performance Expectancy (PE)	PE1	mHealth apps are helpful for my health behaviors.	[27]
	PE2	mHealth apps solve my health questions.	
	PE3	mHealth apps manage my health quickly.	
	PE4	mHealth apps increase my capability of health self-management.	
Effort Expectancy (EE)	EE1	I learn to use mHealth apps easily.	[27]
	EE2	I know lots of health information through mHealth apps.	
	EE3	I use mHealth apps easily.	
	EE4	I get using mHealth apps skill.	
Social Influence (SI)	SI1	I adopt mHealth apps based on friends' perspectives.	[27]
	SI2	I adopt mHealth apps based on peoples' who affect my behavior.	
	SI3	I adopt mHealth apps based on my friends.	
	SI4	Using mHealth apps is more prestigious than not using them.	
Facilitating Conditions (FC)	FC1	I possess the resources needed to adopt mHealth apps.	[27]
	FC2	I possess the knowledge needed to adopt mHealth apps.	
	FC3	The technologies of adoption is consistent with my others.	
	FC4	I gain lots of helps from other users when I account for problems.	
Cognitive Trust (CT)	CT1	I get exact and true health information in mHealth apps.	[58]
	CT2	I depend on the health information through mHealth apps.	
	CT3	mHealth apps are reliable.	
	CT4	mHealth apps keep criterion constantly.	
Online Rating (ORT)	ORT1	Online ratings are believable.	[34,60]
	ORT2	Online ratings are relevant to my demanded.	
	ORT3	Online ratings are trust.	
	ORT4	Online ratings make me perceived reliability.	
	ORT5	Online ratings provided in mHealth apps have helped me to learn about the healthcare services.	
	ORT6	Online ratings provided in mHealth apps have improved my understanding of the healthcare services.	
	ORT7	Online ratings provided in mHealth apps were useful to evaluate the quality of healthcare services.	
Altruism (AL)	AL1	I like to help other users in the mHealth apps.	[8]
	AL2	I enjoy helping others in the mHealth apps.	
	AL3	I feel happy to support others in the mHealth apps.	
Habit (HB)	HB1	The use of mHealth apps turn into my custom.	[27]
	HB2	I am immersed in accepting mHealth apps.	
	HB3	I have to adopt mHealth apps.	
	HB4	I think the adoption of mHealth apps have turn into become intrinsic behavior.	
Behavioral Intention (BI)	BI1	I prepare to continue accepting these platforms in the future.	[27]
	BI2	I always accept these platforms in daily life.	
	BI3	I purpose to continue accepting these platforms frequently.	

Usage Behavior (UB)	UB1	I go through much time in using these platforms.	[27]
	UB2	I indeed desire these platforms to keep my healthcare safe.	
	UB3	I use these platforms on regular basis.	

Note: CR = Composite Reliability, AVE = Average Variance Extracted, PE = Performance Expectancy, EE = Effort Expectancy, SI = Social Influence, AL = Altruism, FC = Facilitating Conditions, HB = Habit, CT = Cognitive Trust, ORT = Online Rating, BI = Behavioral Intention, UB = Usage Behavior.

Appendix B. Results of exploratory factor analysis.

Items	Component									
	1	2	3	4	5	6	7	8	9	10
PE1	0.098	0.170	0.140	0.054	**0.787**	0.357	0.045	0.034	0.382	0.078
PE2	0.281	0.012	0.077	0.215	**0.782**	0.173	0.107	0.262	0.155	0.340
PE3	0.194	0.102	0.151	0.072	**0.792**	0.141	0.087	0.042	0.023	0.128
PE4	0.189	0.145	0.228	0.077	**0.805**	0.069	0.242	0.157	0.140	0.040
EE1	0.108	**0.734**	0.041	-0.005	-0.015	0.196	0.102	0.145	0.257	-0.002
EE2	0.198	**0.727**	0.158	0.231	0.221	0.123	0.245	0.272	-0.028	0.043
EE3	0.154	**0.795**	0.131	0.040	0.013	0.216	0.080	0.020	0.273	0.155
EE4	0.163	**0.715**	0.079	0.058	0.220	0.061	0.269	-0.035	-0.047	0.075
SI1	0.198	0.112	**0.777**	0.172	0.199	0.141	0.064	0.097	0.185	0.032
SI2	0.120	0.174	**0.757**	0.209	0.130	0.218	0.063	0.114	-0.035	0.090
SI3	0.215	0.051	**0.739**	0.201	0.140	0.090	0.122	0.197	0.064	0.067
SI4	0.065	0.007	**0.710**	0.371	-0.036	0.040	0.251	0.431	0.135	0.108
AL1	0.032	0.348	0.061	0.100	0.166	0.006	0.099	-0.011	**0.795**	0.270
AL2	0.075	0.214	0.091	0.217	0.208	0.184	0.194	0.336	**0.734**	0.034
AL3	0.172	0.307	0.093	0.086	0.181	0.077	0.389	0.209	**0.871**	-0.012
FC1	0.263	0.206	0.078	0.096	0.061	0.123	0.223	0.110	0.061	**0.812**
FC2	0.272	0.077	0.208	0.030	0.042	0.221	0.172	0.129	0.018	**0.745**
FC3	0.163	0.137	0.152	0.234	0.113	-0.012	0.115	0.248	0.005	**0.758**
FC4	0.102	0.080	0.055	0.148	0.144	0.071	0.057	0.115	0.150	**0.736**
HB1	0.294	0.138	0.278	0.058	0.134	0.261	**0.805**	0.009	0.119	0.175
HB2	0.193	0.306	0.126	0.216	0.311	0.426	**0.728**	0.058	0.226	-0.048
HB3	0.219	0.036	0.231	0.128	0.213	0.469	**0.847**	0.007	0.163	0.161
HB4	0.216	0.257	0.205	0.154	0.276	0.373	**0.839**	0.058	0.084	0.071
CT1	0.156	0.249	0.251	**0.864**	0.170	0.175	0.072	0.139	-0.015	-0.002
CT2	0.217	0.225	0.156	**0.719**	0.182	0.035	0.168	0.085	0.074	0.353
CT3	0.218	0.094	0.173	**0.757**	0.184	0.232	0.203	0.120	0.087	0.145
CT4	0.134	0.240	0.265	**0.809**	0.168	0.159	0.155	0.119	0.083	0.026
ORT1	0.167	0.256	0.102	0.248	0.243	**0.738**	0.189	0.148	-0.124	0.070
ORT2	0.199	0.251	0.144	0.159	0.057	**0.857**	0.05	-0.125	0.027	0.239
ORT3	0.191	-0.015	0.154	0.244	0.228	**0.906**	0.261	0.023	0.295	0.071
ORT4	0.072	0.117	0.136	0.146	0.003	**0.701**	0.03	0.263	-0.048	0.135
ORT5	0.235	0.074	0.228	0.179	0.048	**0.895**	-0.004	0.062	0.006	0.114
ORT6	0.291	0.098	0.21	0.049	0.173	**0.726**	0.276	-0.009	0.211	-0.016
ORT7	0.169	0.131	0.16	0.009	0.073	**0.921**	0.068	0.145	0.359	-0.027
BI1	0.296	0.094	0.137	0.064	0.009	0.186	0.093	**0.781**	0.156	0.066
BI2	0.145	0.01	0.256	0.254	0.098	0.122	0.109	**0.848**	0.05	0.163
BI3	0.153	0.189	0.185	0.163	0.211	0.099	0.112	**0.841**	0.077	0.183
UB1	**0.843**	0.259	0.061	0.283	0.243	0.088	0.172	0.108	0.037	0.028
UB2	**0.761**	0.238	0.098	0.235	0.195	0.014	0.153	0.116	0.083	0.164
UB3	**0.616**	0.071	0.125	0.089	0.191	0.219	0.273	0.012	0.128	0.053
